# Does complete resection of infected bone improve clinical outcomes in patients with diabetic foot osteomyelitis?

**DOI:** 10.1111/iwj.70072

**Published:** 2024-10-07

**Authors:** Lawrence A. Lavery, Arthur N. Tarricone, Mario C. Reyes, Mehmet A. Suludere, Matthew J. Sideman, Michael C. Siah, Edgar J. G. Peters, Dane K. Wukich

**Affiliations:** ^1^ Department of Plastic Surgery University of Texas Southwestern Medical Center Dallas Texas USA; ^2^ Department of Orthopedic Surgery University of Texas Health Science Center San Antonio Texas USA; ^3^ Department of Surgery University of Texas Health Science Center San Antonio Texas USA; ^4^ Department of Surgery University of Texas Southwestern Medical Center Dallas Texas USA; ^5^ Department of Internal Medicine, Section of Infectious Diseases, Amsterdam UMC Vrije Universiteit Amsterdam Amsterdam The Netherlands; ^6^ Amsterdam Institute for Infection and Immunity and Amsterdam Movement Sciences Amsterdam The Netherlands; ^7^ Department of Orthopedic Surgery University of Texas Southwestern Medical Center Dallas Texas USA

**Keywords:** amputation, diabetes, infection, osteomyelitis, ulcer

## Abstract

The objective of the study was to compare outcomes in patients with complete surgical resection versus partial resection of diabetic foot osteomyelitis (OM). A post hoc analysis of 171 patients with OM was performed using data from two randomized clinical trials. OM was confirmed with bone culture or histopathology. Surgical culture specimens were obtained from resected bone and sent for histopathology and microbiology. Residual osteomyelitis (RO) was defined as a positive resected margin on culture or histopathology. No residual osteomyelitis (NRO) was defined as no growth from bone culture and no histopathological inflammation in the biopsy of the resection margin. Data from the 12‐month follow‐up were used to determine clinical outcomes. During the index hospitalization, NRO patients had significantly shorter duration of antibiotic therapy (NRO 21.0, 13.0–38.0 vs. RO 37.0, 20.8–50.0, *p* <0.01) and more amputations than patients with RO (NRO 89.9% vs. RO 60.9%, *p* <0.01). During the 12‐month follow‐up, patients with NRO also had significantly shorter duration of antibiotic therapy (NRO 42, 21.0–66.5 vs. RO 50.5, 35.0–75.0, *p* = 0.02). During the 12‐month follow‐up, there was no difference in ulceration at the same site (NRO 3.7%, RO 4.3% *p* = 0.85), hospitalization (NRO 32.6%, RO 34.8%, *p* = 0.76), total re‐infections (NRO 25.3%, RO 29.3%, *p* = 0.56), re‐infection with osteomyelitis (NRO 13.3% vs. 13.5%, *p* = 0.36), amputation (NRO 8.8%, RO 5.4%, *p* = 0.86) and time to wound healing in days (NRO 94, 41.0–365 vs. RO 106, 42.8–365, *p* = 0.77). Successful treatment of osteomyelitis was achieved by 86.7% and 86.5% of patients. During the index hospitalization, patients with no residual osteomyelitis had more amputations and were treated with antibiotics for a shorter duration. During the 12‐month follow‐up, patients with no residual osteomyelitis had shorter durations of antibiotics. There were no differences in re‐infection, amputation, re‐ulceration or hospitalization.

**Level of evidence:** 1

## 1 INTRODUCTION

The Infectious Diseases Society of American (IDSA) and International Working Group on the Diabetic Foot (IWGDF)^1^, ^2^ have provided recommendations for the treatment and management of diabetic foot infections. The 2012 IDSA diabetic foot infection guidelines specifically included a section on the duration of antibiotic treatment based on the presence and type of surgical excision of infected bone, even though there was no evidence at the time.^3^ If only part of the infected bone was excised, leading to residual bone infection, 4 weeks of antibiotics was recommended. With complete resection of the infected bone, IDSA guidelines recommend antibiotic treatment for 5 days. In contrast, the IWGDF/IDSA's most recent collaborative guidelines recommend up to 3 weeks of antibiotics after surgery if there are positive bone margins and 6 weeks without bone resection or amputation. If the infected bone is completely resected, antibiotics for 2–5 days are recommended. These recommendations are based on small retrospective studies with low certainty of evidence and high risk of bias.^2^ The goal of this study was to evaluate the clinical outcomes of patients with diabetes and foot osteomyelitis who had complete resection of infected bone and patients with residual bone infection after surgery. We hypothesized that patients with complete excision would have better clinical outcomes and receive shorter duration of antibiotics than patients with residual infected bone.

## 2 METHODS

This study was approved by the Institutional Review Board at our institution. We performed a pooled, patient‐level, post hoc analysis using data from two randomized controlled trials by our group that used the same evaluation criteria and operational definitions for outcomes and adverse events.^4^, ^5^ In the RCTs, we enrolled 240 patients with moderate and severe diabetic foot infections using the criteria defined by IWGDF between the ages of 18 and 89. For this post hoc analysis, we only included diabetic patients with osteomyelitis who had at least 12‐month follow‐up. All of the study subjects underwent surgery for infection. Confirmation of the initial diagnosis of osteomyelitis was made by bone biopsy with either positive bone culture or bone histopathology.^6^ The absence of osteomyelitis was determined by a negative MRI, negative SPECT CT or negative bone biopsy (both histology and culture).^7^, ^8^, ^9^ After the initial incision and drainage or amputation, the surgical site was irrigated with normal saline and bone cultures were obtained from the infected bone. After bone resection, a section of bone was obtained from the proximal margin and sent for culture and histopathology to determine if there was residual bone infection. Successful treatment of osteomyelitis was defined as no bone re‐infection in the year following the index hospitalization.

We documented the history of foot ulceration and amputation of the foot or leg. Sensory neuropathy was defined as abnormal vibration sensation, absence of sensation with a 10‐g Semmes–Weinstein monofilament, or absent Achilles tendon reflexes. Peripheral arterial disease was defined as an ankle to arm systolic blood pressure ratio (ABI) of <0.90. Non‐compressible artery was defined as an ABI greater than 1.30. Foot ulceration was defined as full‐thickness skin lesions involving any portion of the foot or ankle, and foot infection severity was defined according to the criteria of the IWGDF.^10^

For the purposes of this paper, we only evaluated patients with diabetes and osteomyelitis. Patients with OM were divided into two groups: patients with no residual osteomyelitis after surgery (NRO) and those with incomplete excision of infection bone with residual osteomyelitis at the margins (RO). Subjects with RO were considered to have residual bone infection in situ. RO was defined as either a positive bone culture or positive pathology report of the bone cultured from the proximal margin. NRO was defined as negative bone culture and negative pathology report for infection or inflammation of the bone cultured from the proximal margins.^6^

Categorical variables were described as frequency and percentage while continuous variables were reported as mean and standard deviation. Differences in patient characteristics between patients with RO and NRO were calculated using chi‐squared test of homogeneity or Fisher exact test for categorical variables. Mann–Whitney *U* test was applied for non‐normally distributed data, and findings were reported as median (IQR). Relative risk was used to evaluate the relationship between bone margin, reinfection and healing. Survival time to event analysis using Kaplan–Meir plot was censored at 365 days for healing and reinfection.

## 3 RESULTS

A total of 240 patients with moderate and severe foot infections were included in the database. This study included 171 patients with diabetes and osteomyelitis (73.4%). Among patients with osteomyelitis, 79 patients (46.2%) had negative margins and 92 patients (53.8%) had positive margins. There were no significant differences between the two groups with regard to age, gender, BMI, social history, medications, retinopathy, peripheral neuropathy, peripheral artery disease, chronic kidney disease, glycated haemoglobin, CRP, WBC, ESR or initial wound volume (Table 1).

**TABLE 1:** Demographics and co‐morbidities in patients with residual osteomyelitis and no residual osteomyelitis.

	No residual osteomyelitis (*n* = 79)	Residual osteomyelitis (*n* = 92)	RR (95% CI)	*P* value
Age	52.4 (8.9)	50.9 (10.5)	−1.5–4.4	0.33
Male	66 (83.5)	72 (78.3)	1.41 (0.7–3.1)	0.38
BMI (kg/m^2^)	31.2 (7.2)	31.5 (7.9)	−2.7–1.9	0.75
Race				
Non‐Hispanic White	19 (24.1)	18 (19.6)	1.3 (0.6–2.7)	0.48
African descent	27 (34.2)	27 (29.3)	1.3 (0.6–2.4)	0.49
Hispanic	31 (39.2)	47 (51.1)	0.6 (0.3–1.1)	0.12
Native American	2 (2.5)	0 (0.0)	2.2 (1.9–2.6)	0.13
Social factors				
<12 years education	27 (34.2)	37 (40.2)	1.2 (0.6–2.2)	0.67
Spanish language	13 (16.4)	21 (22.8)	0.7 (0.3–1.6)	0.42
Living alone	10 (12.7)	12 (13.0)	1.0 (0.4–2.6)	0.93
Household ambulator	1 (1.3)	5 (5.4)	0.2 (0.0–1.7)	0.11
Married	31 (39.2)	36 (39.21)	1.0 (0.5–1.9)	0.98
Current tobacco use	16 (20.3)	20 (21.7)	0.9 (0.4–1.9)	0.81
Current alcohol use	22 (27.8)	19 (20.7)	1.5 (0.7–3.0)	0.27
Current illicit drug use	4 (5.1)	4 (4.3)	1.2 (0.3–4.9)	0.83
Medical history				
MI	6 (7.6)	5 (5.4)	1.4 (0.4–4.8)	0.56
CHF	9 (11.4)	10 (10.9)	1.1 (0.4–2.7)	0.91
HIV	2 (2.5)	0 (0.0)	2.2 (1.8–2.6)	0.13
Retinopathy	11 (13.9)	19 (20.7)	0.6 (0.3–1.4)	0.25
CKD I‐IV	17 (18.5)	23 (29.1)	1.8 (0.7–3.8)	0.10
ESRD	7 (8.8)	4 (4.3)	2.1 (0.6–7.6)	0.23
Monckeberg's sclerosis	43 (54.4)	56 (60.9)	0.8 (0.4–1.4)	0.39
Sensory neuropathy	76 (97.4)	90 (97.8)	0.8 (0.1–6.1)	0.87
Abnormal monofilament	67 (87.0)	82 (91.1)	0.7 (0.2–1.7)	0.39
VPT forefoot	50.1 (25.1)	48.8 (22.4)	−5.9–8.5	0.73
Charcot arthropathy history	4 (5.1)	4 (4.3)	1.2 (0.3–1.1)	0.83
Prior foot ulcer	49 (62.0)	67 (72.8)	0.6 (0.3–1.2)	0.13
Prior amputation	33 (41.8)	50 (54.3)	0.6 (0.3–1.1)	0.10
Medications				
Insulin	61 (66.3)	50 (63.3)	0.9 (0.5–1.6)	0.68
Corticosteroids	3 (3.8)	11 (11.9)	0.29 (0.08–1.1)	0.07
Calcium channel blockers	20 (25.3)	24 (26.1)	0.09 (0.5–1.9)	0.91
Beta‐blockers	14 (17.7)	23 (25.0)	0.6 (0.3–1.4)	0.25
Gabapentin	21 (26.6)	31 (33.7)	0.7 (0.4–1.4)	0.31
Pre‐gabalin	2 (2.5)	5 (5.4)	0.5 (0.1–2.4)	0.35
Admission characteristics				
SIRS criteria	19 (24.1)	17 (18.5)	1.4 (0.7–2.9)	0.37
Temperature >38.6	10 (12.7)	11 (12.0)	1.1 (0.4–2.7)	0.89
Heart rate >90	29 (36.7)	39 (42.4)	0.7 (0.4–1.5)	0.45
Respiratory rate >20	17.5 (1.7)	17.7 (2.1)	1.5 (0.6–4.1)	0.50
WBC >12 000	25 (31.6)	27 (29.7)	1.1 (0.5–2.1)	0.78
WBC	10.8 (4.5)	10.7 (4.1)	−1.2–1.3	0.94
Admissions labs				
CRP	10.8 (11.3)	12.3 (11.5)	−5.0–1.9	0.37
ESR	75.6 (37.6)	80.4 (41.8)	−16.7–7.6	0.46
Glycated haemoglobin	9.5 (2.7)	9.6 (2.6)	−0.9–0.6	0.71
eGFR	51.0 (16.3)	53.1 (14.5)	−6.7–2.5	0.37
Wound characteristics				
Ulcer duration (days) Median (IQR)	29 (10–60)	30 (14–90)		0.22
Wound area (cm^2^) Median (IQR)	11.4 (6.8–24.0)	10.7 (5.5–17.6)		0.19
Wound volume (cm^3^) Median (IQR)	9.1 (3.8–23.8)	7.4 (2.2–16.3)		0.15
Ankle brachial index				
<0.90	11 (13.9)	6 (6.6)	2.3 (0.8–6.5)	0.11
0.90–1.30	45 (57.0)	57 (62.6)	0.8 (0.4–1.5)	0.45
>1.30	23 (29.1)	28 (30.8)	0.9 (0.5–1.8)	0.81
Toe brachial index	0.71 (0.3)	0.75 (0.3)	−0.1–0.1	0.40

*Note*: Dichotomous variables presented as *N* (%). Continuous variables presented as either mean (standard deviation) or median (IQR) for non‐normally distributed data.

Abbreviations: CRP, C‐reactive protein; eGFR, estimated glomerular filtration rate; ESR, erythrocyte sedimentation rate; VPT, vibration perception threshold testing; WBC, white blood cell count.

During the index hospitalization, patients with negative bone margins had significantly shorter duration of antibiotic therapy (NRO 21.0 days, 13.0–38.0 vs. RO 37.5, 13.0–38.0, *p* <0.01) and significantly more amputations than patients with RO (NRO 89.9% vs. RO 60.9%, *p* <0.01). There was no difference in the length of hospitalization (NRO 12.0, 9.0–17.5 vs. RO 13.0, 0.0–16.5).

**FIGURE 1:**
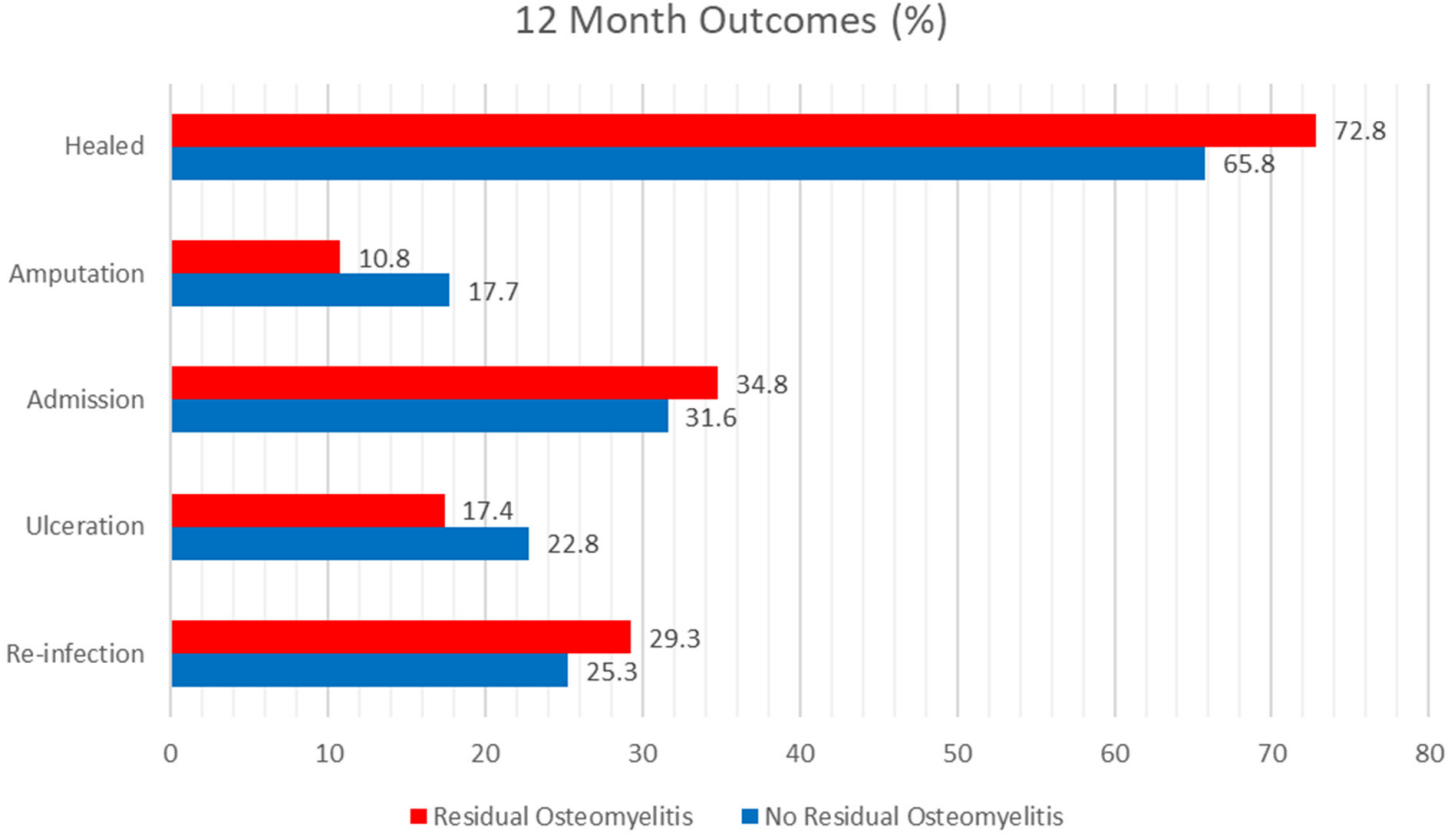
Twelve‐month outcome comparison of patients with residual osteomyelitis and no residual osteomyelitis. This bar chart compares the 12‐month clinical outcomes in patients with residual osteomyelitis and no residual osteomyelitis. There were no differences in clinical outcomes after hospital discharge.

During the 12‐month follow‐up, there was no difference in the incidence of healing (NRO 65.8% vs. RO 72.8%, *p* = 0.99), re‐infection with osteomyelitis (NRO 13.3% vs. RO 13.5%, *p* = 0.36), amputation (NRO 17.7% vs. RO 10.8%, *p* = 0.76), hospitalization (NRO 30.4%, RO 32.6%, *p* = 0.76), length of hospitalization (NRO 12.0, 9.0–17.5 vs. RO 0.0, 10.0–16.5), time to wound healing in days (NRO 94.0, 41.0–365 vs. RO 106.0, 42.8–365, *p* = 0.77) and ulceration on the same site (NRO 3.7% vs. RO 4.3% *p* = 0.85) (Figure 1).

**FIGURE 2:**
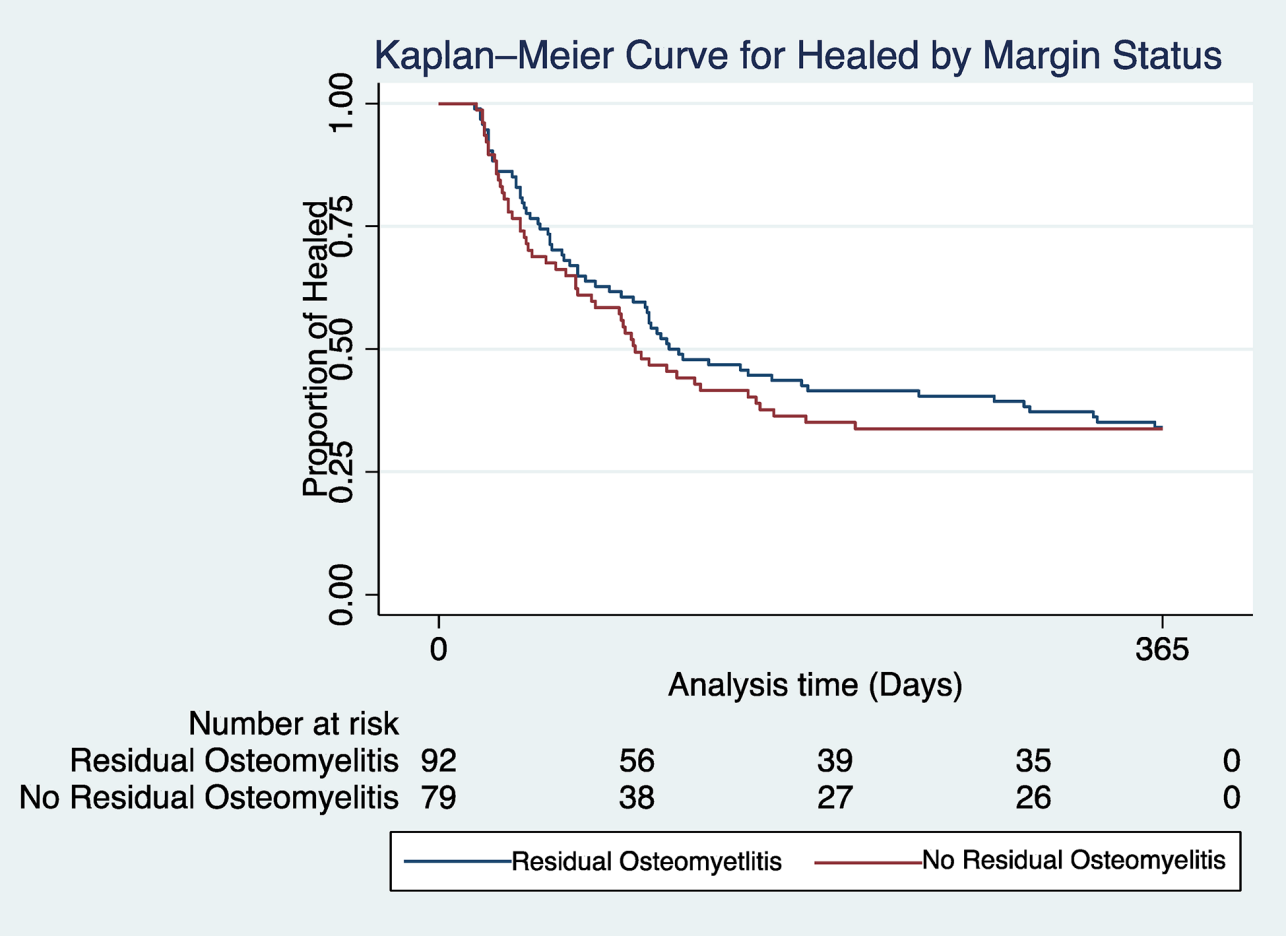
Kaplan–Meier survival plot comparing the time to heal in patients with residual osteomyelitis and no residual osteomyelitis. Kaplan–Meier survival analysis of reinfection by margin status. The *Y*‐axis represents the proportion healed, and the *X*‐axis represents the study period (days). The comparison in re‐infection in patients with residual osteomyelitis and no residual osteomyelitis demonstrates that there is no significant difference in time to healing between the two groups. The average time until healing for NRO = 73.7 ± 61.4 versus RO = 75.4 ± 65.1.

**FIGURE 3:**
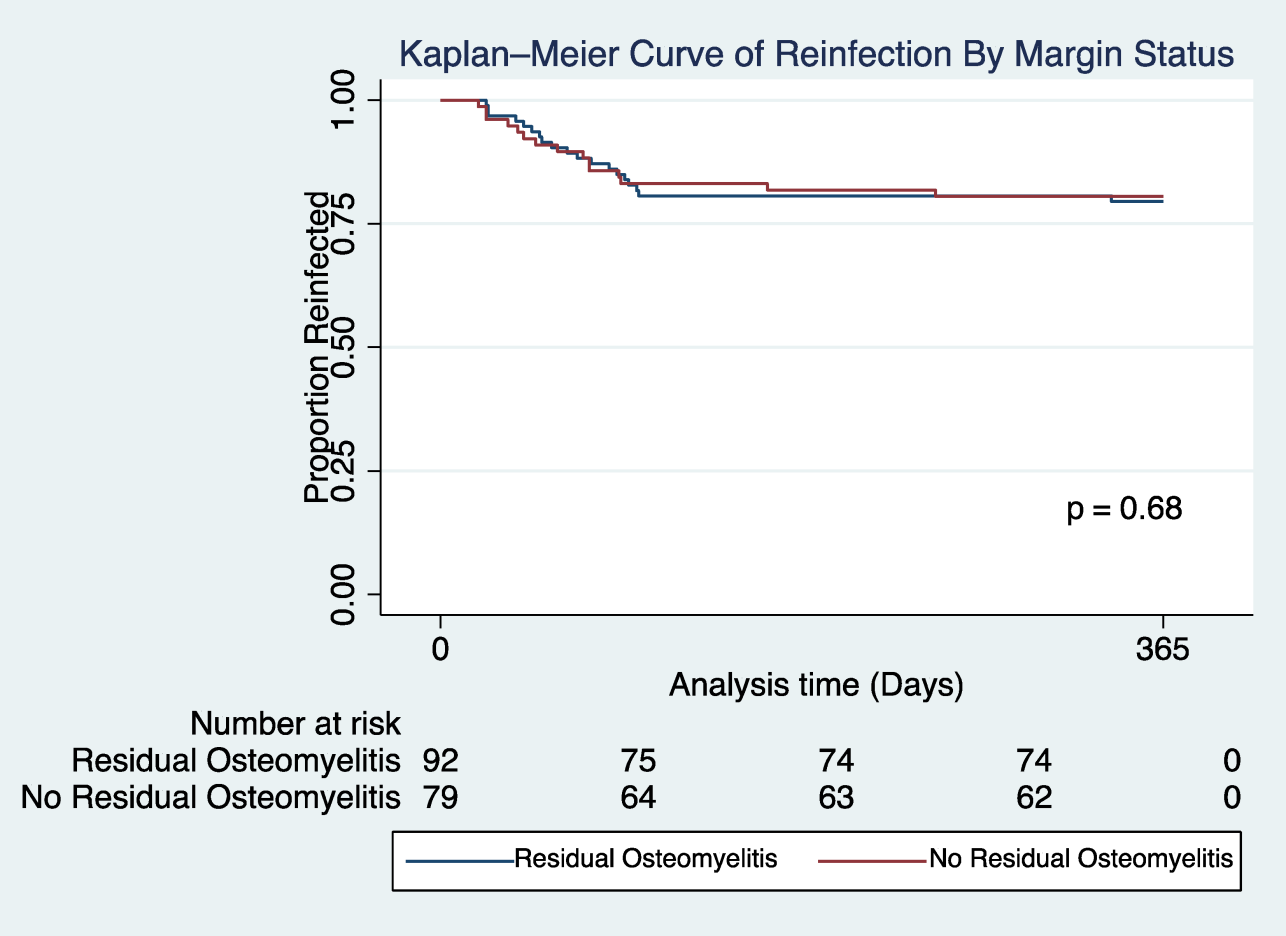
Kaplan–Meier survival analysis of reinfection by margin status. The *Y*‐axis represents the proportion without reinfected, and the *X*‐axis represents the study period (days). The comparison in re‐infection in patients with residual osteomyelitis and no residual osteomyelitis demonstrates that there is no significant difference in time to re‐infection between the two groups. The average time until healing for RO was 98.7 ± 86.6 and NRO = 72.8 ± 49.4. Kaplan–Meier survival analysis comparing the time to re‐infection in patients with a residual osteomyelitis and no residual osteomyelitis in demonstrates that there is no significant difference in time to re‐infection between the two groups.

There was a significantly shorter duration of antibiotic therapy in people with no residual osteomyelitis compared to patients with RO (NRO 42.0, 21.0–66.5 vs. RO 50.5 35.0–75.0, *p* <0.02; Table 2). In the Kaplan–Meier survival analysis, there was no difference in the time to wound healing in days (NRO 73.7 ± 61.4 vs. RO 75.4 ± 65.3, *p* = 0.06) and time to reinfection (NRO 72.8 ± 49.4 vs. RO 98.7 ± 86.6, *p* = 0.93) in patients with NRO and RO (Figures 2 and 3).

**TABLE 2:** Clinical outcomes in patients with residual osteomyelitis and no residual osteomyelitis.

	No residual osteomyelitis (*n* = 79)	Residual osteomyelitis (*n* = 92)	RR (CI)	*P*‐value
Index hospital admission				
Amputation	71 (89.9)	56 (60.9)	5.7 (2.5–13.2)	**0.001**
Length of stay (days) Median (IQR)	12.0 (9.0–17.5)	13.0 (10.0–16.5)		0.29
Antibiotic days Median (IQR)	21.0 (13.0–38.0)	37.5 (20.8–50.0)		**0.001**
12‐month follow‐up period				
Healed	52 (65.8)	67 (72.8)	0.8 (0.4–1.5)	0.47
Time to heal (days) Median (IQR)	94.0 (41.0–365)	106.0 (42.8–365)		0.77
Re‐infection	20 (25.3)	27 (29.3)	0.8 (0.4–1.6)	0.56
Re‐infection—soft tissue	8 (9.6)	8 (16.9)	1.2 (0.4–3.3)	0.75
Re‐infection—bone	12 (13.3)	19 (13.5)	0.7 (0.3–1.5)	0.36
Ulceration—total	21 (26.6)	20 (21.7)	1.3 (0.6–2.6)	0.46
Ulceration—same site	3 (3.7)	4 (4.3)	0.9 (0.2–4.0)	0.85
Ulceration—other sites	18 (22.8)	16 (17.4)	1.4 (0.6–2.9)	0.37
Hospital admission	25 (31.6)	32 (34.8)	0.9 (0.5–1.7)	0.66
Amputation	14 (17.7)	10 (10.8)	1.8 (0.7–4.2)	0.20
Foot amputation	7 (8.8)	5 (5.4)	0.7 (0.3–1.6)	1.0
Leg amputation	7 (8.8)	5 (5.4)	0.7 (0.3–1.6)	1.0
Total length of stay (days) Median (IQR)	16.0 (10.0–27.0)	15.0 (11.0–23.3)		0.83
Total antibiotic days Median (IQR)	42.0 (21.0–66.5)	50.5 (35.0–75.0)		**0.02**

*Note*: Descriptive variables are presented as *N* (%). Duration is presented as days. Outcomes are clinically collected within a year after initial surgical treatment. Bold values are statistically significant.

Successful treatment of osteomyelitis varied considerably based on the outcome measure used to define osteomyelitis remission. Our primary outcome to define successful treatment was bone re‐infection. There was no difference in reinfection based on RO or NRO (13.3% vs. 13.5%, *p* = 0.36). Other clinical outcomes have been used to define treatment success for osteomyelitis. None of these parameters were significant. There was no difference in the incidence of healing (NRO 65.8% vs. RO 72.8%, *p* = 0.99), re‐infection (NRO 5.3% vs. RO 29.3%, *p* = 0.56) and amputation after the index hospitalization (NRO 17.7% vs. RO 10.8%, *p* = 0.76) in patients with negative bone margins and patients with residual osteomyelitis.

## 4 DISCUSSION

The results suggest that despite differences in surgical approaches and antibiotic treatment during the index hospitalization, no differences in 12‐month outcomes for patients with diabetic foot osteomyelitis were observed between those who had NRO and RO (residual osteomyelitis). During the index hospitalization, NRO patients underwent significantly higher rates of amputation. Intuitively, this makes sense since amputation allows all infected bones to be removed. Ultimately, the decision for antibiotic duration is typically made by the surgeon, infectious disease specialist and the patient. Although amputation is more likely to provide a clean bone margin, foot amputations can result in biomechanical derangement of the foot and an increased risk of ulcers and poor healing.^11^, ^12^

Patient preference certainly plays a major role in surgical decisions. A recent study reported that patients with diabetic foot disease fear major amputation, minor amputation and foot infection more than death, blindness, heart attack, stroke and dialysis.^13^, ^14^ Consequently, many patients are averse to proceeding with minor amputation despite requiring prolonged intravenous and/or oral antibiotic therapy. In contrast, surgeons may have a bias towards amputation to achieve complete resection of infected bone and often do not believe osteomyelitis can be treated successfully with antibiotics. The data from this study did not demonstrate a difference in outcomes between the two approaches, and consequently, providers can consider non‐ablative surgical options in this group of patients without differences in 12‐month outcomes. Specifically, there was no difference in wound healing, re‐infection, hospitalization and subsequent amputation during the 12‐month follow‐up. To our knowledge, this is the first prospective study that evaluates how clinical outcomes are related to the presence or absence of residual bone infection.

The most recent diabetic foot guidelines are a collaboration of the IWGDF and IDSA.^3^, ^6^ The IDSA and IWGDF recommendations are mostly based on expert opinion, since there are only a few studies which classify residual osteomyelitis, colloquially termed clean bone margins and report outcomes based on the presence of residual osteomyelitis. In contrast with our results, other studies report significantly better clinical outcomes in people with complete resection of the infected bone with the demonstration of clean margins. Studies by Kowalski, Atway and Hachmoller report better clinical outcomes when there is evidence that all of the infected bone had been surgically excised. Kowalski et al.^15^ report the results of a retrospective series of 111 patients with osteomyelitis in which negative bone margins, or evidence that all bone infection had been excised, are associated with a lower rate of treatment failure, a shorter duration of antibiotic therapy, lower rate of leg amputations and fewer re‐infections. Hachmoller^16^ report the results of a retrospective study of 45 patients with osteomyelitis with 54 infected bones. Patients with negative bone margins had a higher proportion of healed wounds (55.0% vs. 8.8%, *p* = 0.003). Likewise, in a retrospective study of 27 patients with osteomyelitis, Atway et al.^17^ report that 41% of patients have residual osteomyelitis. Patients with a negative bone margin had significantly fewer complications and better clinical outcomes, compared with patients with a residual infection in the bone (81.8 vs. 25.0%, *p* = 0.006). These findings contrast with our results, which showed no difference in clinical outcomes after the index hospitalization.

Weng et al. evaluated the correlation between resection margins and the 1‐year clinical outcomes. This retrospective study of 92 patients with diabetes and osteomyelitis demonstrated that patients with a positive margin had worse outcomes and required more amputation within 12 months. In contrast to our study, Weng et al. only used histopathology criteria to diagnose OM, potentially leading to under and overestimation of residual OM. They reported that patients with no residual bone infection received antibiotics for a shorter period of time than patients with residual bone infection.^18^

There are important limitations to this study. Most retrospective studies on treatment of osteomyelitis use surrogate outcomes to define successful treatment of osteomyelitis, such as wound healing, recurrent foot ulceration or amputation.^19^, ^20^, ^21^, ^22^, ^23^, ^24^, ^25^ While some of these factors may be more common in people with diabetes, there is not a direct relationship with any of these outcomes and bone infections. For instance, the chance of ulcer recurrence is 30%–40% within 12 months in people with a healed diabetic foot ulcer with no history of either a bone or soft tissue infection.^26^ Likewise, there are low rates of wound healing (30% in 12 weeks) in small full thickness diabetic foot ulcers with no history of bone infection. Osteomyelitis is not a risk factor for poor wound healing.^27^, ^28^ And while there may be higher rates of amputation in people with osteomyelitis, infection and amputation are also common in people with soft tissue infections.^29^, ^30^ Surrogate markers are not specific for osteomyelitis and are poor tools to evaluate the treatment success or failure. Ideally, more direct measures of bone activity such as MRI or bone biopsy would be used to determine if infection has been treated successfully. However, these are rarely performed to monitor the success of treatment in patients with osteomyelitis in both clinical practice and research settings. While we did not find a difference in our total length of stay (days), this may reflect the nature of our patient population. Our primary hospital is a large inner city, safety net hospital and discharge planning is quite difficult.

The duration of treatment for osteomyelitis varies widely in reported clinical studies.^31^ There is a possibility of selection bias and confirmation bias in the studies identified in this systematic review, because the treating physician may have based the duration of antibiotic on the positive or negative bone margin status. The decision to treat with antibiotics for longer or shorter duration can be influenced by adherence to the recommendations of the IDSA/IWGDF diabetic foot infection guidelines or on dogmas to treat osteomyelitis for a set period, rather than to use clinical, laboratory, or imaging criteria as stopping rules for antibiotics. Patients with no residual infection after surgical resection are increasingly being used to identify that all the infected bone has been excised. Proof of a no residual bone infection should make further antibiotic or surgical therapy unnecessary. This is the approach that the 2012 IDSA guideline first made when they recommended 2–5 days of additional antibiotics after surgery with a no residual bone infection.^3^ The latest collaborative guidelines by the IWGDF and IDSA make similar recommendations.^2^ Our data would suggest that this is not yet the case in clinical practice. While patients with residual bone infection received antibiotics longer (58.9 days) than patients with no residual infection (49.9 days), the duration of treatment looked more like the traditional 6–7 weeks (49.9 days) rather than the abbreviated course of oral therapy that has been recommend.

Another limitation of the current study is that different infectious disease specialists were usually responsible for antibiotic treatment decisions including the route, type and duration of antibiotic therapy. As there is no agreed upon algorithm for treatment of osteomyelitis, each infectious disease provider may have used different definitions of clinical remission and thus varying ‘stopping rules’ for ending therapy. Certainly, some providers undoubtedly relied on the traditional dogma of 6 weeks of antibiotic therapy for osteomyelitis. The major strength of this study compared to previously published literature is its size and robust operational definition of the diagnosis of osteomyelitis.

## 5 CONCLUSION

We did not find any difference in clinical outcomes or post‐operative complications or duration of antibiotic treatment in patients that had complete surgical resection of infected bone compared with patients with residual osteomyelitis.

## FUNDING INFORMATION

This work was supported by the American Diabetes Association (Translational Research Award 7‐14‐TS‐20) and Cardinal Health.

## CONFLICT OF INTEREST STATEMENT

The authors declare no conflicts of interest.

## Data Availability

The data that support the findings of this study are available from the corresponding author, upon reasonable request.
